# Trends in Atrial Fibrillation Management—Results from a National Multi-Center Urgent Care Network Registry

**DOI:** 10.3390/jcm12216704

**Published:** 2023-10-24

**Authors:** Shalom Lebovitz, Menachem Estryk, Deena R. Zimmerman, Arthur Pollak, David Luria, Offer Amir, Yitschak Biton

**Affiliations:** 1 Department of Cardiology, Hadassah Medical Center, Faculty of Medicine, Hebrew University of Jerusalem, Jerusalem 91904, Israeloamir@hadassah.org.il (O.A.); 2 TEREM—Emergency Medical Centers, Jerusalem 97775, Israel

**Keywords:** atrial fibrillation, urgent care center, urgent care clinic, walk-in clinic, emergency department, UCC

## Abstract

Background: Atrial fibrillation (AF) is a common diagnosis in patients presenting to urgent care centers (UCCs), yet there is scant research regarding treatment in these centers. While some of these patients are managed within UCCs, some are referred for further care in an emergency department (ED). Objectives: We aimed to identify the rate of patients referred to an ED and define predictors for this outcome. We analyzed the rates of AF diagnosis and hospital referral over the years. Finally, we described trends in patient anticoagulation (AC) medication use. Methods: This retrospective study included 5873 visits of patients over age 18 visiting the TEREM UCC network with a diagnosis of AF over 11 years. Multivariate analysis was used to identify predictors for ED referral. Results: In a multivariate model, predictors of referral to an ED included vascular disease (OR 1.88 (95% CI 1.43–2.45), *p* < 0.001), evening or night shifts (OR 1.31 (95% CI 1.11–1.55), *p* < 0.001; OR 1.68 (95% CI 1.32–2.15), *p* < 0.001; respectively), previously diagnosed AF (OR 0.31 (95% CI 0.26–0.37), *p* < 0.001), prior treatment with AC (OR 0.56 (95% CI 0.46–0.67), *p* < 0.001), beta blockers (OR 0.63 (95% CI 0.52–0.76), *p* < 0.001), and antiarrhythmic medication (OR 0.58 (95% CI 0.48–0.69), *p* < 0.001). Visits diagnosed with AF increased over the years (*p* = 0.030), while referrals to an ED decreased over the years (*p* = 0.050). The rate of novel oral anticoagulant prescriptions increased over the years. Conclusions: The rate of referral to an ED from a UCC over the years is declining but remains high. Referrals may be predicted using simple clinical variables. This knowledge may help to reduce the burden of hospitalizations.

## 1. Introduction

Urgent care clinics (UCCs) are a type of health facility located in communities that provide ambulatory care for patients, usually in a walk-in fashion and during out-of-hours service. Different UCC models exist worldwide with a range of primary care and emergency medicine capabilities [1,2,3]. UCCs reduce inappropriate ED referrals by improving the triage of patients [4,5]. Studies from Israel, Ireland, and New Zealand have shown a 35–40% reduction in ED visits per capita in areas with an established UCC [1,6].

Atrial fibrillation (AF) is the most common chronic arrhythmia in the adult population. AF is associated with increased all-cause mortality, stroke, congestive heart failure, disabling symptoms, and reduced quality of life [7,8]. Contemporary treatment includes anticoagulation (AC), rate or rhythm control through pharmacotherapy, ablation, and cardioversion [9]. Previous studies have demonstrated that the rate of emergency department (ED) visits and hospitalizations due to AF is consistently increasing [10,11]. Currently, there is a paucity of information on whether UCCs contribute to reducing the burden of AF patients in EDs.

The new ESC guidelines emphasize the holistic approach in treating patients with AF, as highlighted in the ABC pathway [12,13]. AC is considered a cornerstone in the management of AF to prevent embolic complications, particularly stroke. Current guidelines recommend NOAC therapy rather than warfarin for patients with non-valvular AF and a CHA_2_DS_2_-VASc score > 1 for men or >2 for women [9,13]. Prescription of anticoagulants in EDs has been shown to be feasible and safe and may contribute to improved survival [14]. Despite this, it was previously shown that the rate of AC prescription at discharge from EDs was insufficient [15,16]. There are no data regarding the rate of AC prescription in UCCs, and it is unknown whether patients with diagnosed AF presenting at these centers are receiving adequate AC at presentation.

The rate of AF diagnosis has been rising worldwide due to the aging population and subsequent increased comorbidities. In addition, it reflects increased utilization of implantable devices and wearable technology in patients who otherwise remain undiagnosed [17,18,19]. While the trends of patients with AF arriving in EDs have been described [20,21], there are no data regarding the diagnostic and treatment trends in patients presenting to UCCs.

In this study, we analyzed the trends of AF diagnosis, treatment, and ED referrals in the TEREM UCC registry over eleven years. Second, we identified predictors associated with referral to an ED. Finally, we described trends in AC therapy at both presentation and discharge.

## 2. Materials and Methods

### 2.1. Study Design

TEREM is a large Israeli multi-center network providing UCC and health services in Israel with approximately 1 million visits per year. All citizens of Israel are insured by a health maintenance organization (HMO) under the power of the national health insurance law (1994). A patient can choose or be referred to either a UCC or ED during out-of-hours service. At discharge from a UCC, immediate primary physician follow-up is recommended, often followed by referral to specialist outpatient care [3].

This study is a retrospective analysis using data from the TEREM Emergency Clinics registry.

### 2.2. Study Population

**Figure 1:**
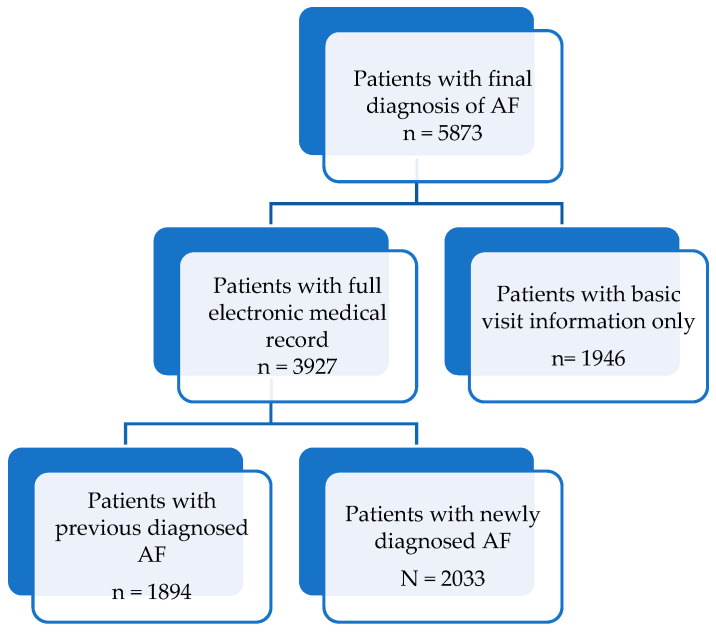
Flow chart of patient population, selection, and subgrouping as used in this study. AF, atrial fibrillation.

We included patients who presented to a UCC between 1 January 2010 and 31 December 2021 and who had a final diagnosis of AF. Only patients who were 18 years or older were included in the study. AF diagnosis was based on the treating physician’s electrocardiography (ECG) interpretation. There were 5873 visits meeting the inclusion criteria, of which 3927 had electronic medical records (Figure 1).

We checked the rate of missing data in pre-specified subgroups to ensure that it was random. Missing data were equally distributed in weekdays, months, shifts, age groups, and sex (all *p*-values were above 0.05).

### 2.3. Variables

We evaluated demographic and clinical variables in the data collection either as continuous or categorial variables. The CHA_2_DS_2_-VASc score was calculated for all study patients based on comorbidities [22]. Also, we calculated a comorbidity score for each patient by adding the numbers of comorbidities (CAD, CHF, CKD, COPD, CVA/TIA, DM, HTN, and PVD) and classified the patients into 3 groups; 0, 1, and 2 or more comorbidities. Antiarrhythmic medication was defined as propafenone, amiodarone, flecainide, sotalol, or digoxin. Anticoagulation medication was defined as apixaban, dabigatran, rivaroxaban, or VKA.

### 2.4. Outcomes

The primary outcome measured was a referral to an ED.

The secondary outcome was the rate of prescribed AC in patients with newly diagnosed AF.

### 2.5. Statistical Analysis

Categorical variables were tested using the chi-square or Fisher’s exact test to determine their association with ED referral. Continuous variables were compared using the *t*-test. Multivariate logistic regression analysis was performed using the backward-elimination-model method to identify predictors of referral to an ED and assess an odds ratio (OR). All relevant clinical variables were considered in the multivariate model (Table A1).

### 2.6. Trends Analysis

We analyzed specific trends over time (categorized by year): (1) number of AF diagnoses per year, (2) AF diagnosis proportion within all UCC visits, (3) percentage of AF visits that were referred to the ED, (4) AC rates. For trend analysis of AF diagnosis and referral to an ED, we used the original sample group (*n* = 5873). The Mantel–Haenszel test for linear trends was used to determine the significance of the trends. All statistical computations were performed using the SPSS software, version 24.0 (IBM). A *p*-value < 0.05 was considered significant.

### 2.7. Subgroup Analyses

We performed a subgroup analysis for patients presenting with a new diagnosis of AF (*n* = 2033).

Trend analysis of AC therapies over the years was performed on patients with a previous diagnosis of AF (*n* = 1894).

## 3. Results

### 3.1. Subject Characteristics

During a period of 11 years, there were 5873 visits with a final diagnosis of AF, of which 3927 patients had full EMR data. Patient characteristics are described in Table 1. The median age was 68.74 years, and 46.8% were male. The mean CHA_2_DS_2_-VASc score was 2.23. The most common presenting symptom was chest pain, followed by palpitations and malaise (25.54%, 23.15%, and 10.65%, respectively).

In UCCs, 36.4% of patients were treated with intravenous verapamil and 8.7% with intravenous amiodarone, and 2.1% underwent cardioversion. Overall, 72.2% of all patients were referred to an ED, of which 56% were transported by an EMS.

Patients referred to an ED were younger and more likely to be male with comorbidities, including hypertension, diabetes, and vascular disease. The group of patients who were discharged home had a higher proportion of previous AF diagnosis, AC, and antiarrhythmic medications.

Patients in the ED group arrived more often during evening or night shifts, had lower SpO_2_, and had higher body temperature. CHA_2_DS_2_-VASc scores were similar between the groups, whereas comorbidity scores were significantly higher in the ED group.

### 3.2. Predictors of ED Referral

**Figure 2:**
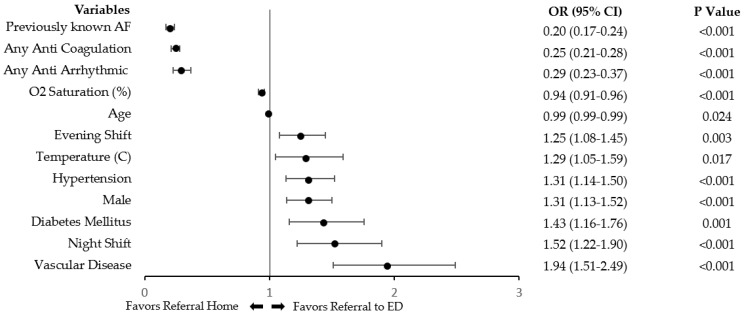
Plot listing pre–specified significant variables in the univariate analysis predicting referral to ED. Black dots represent OR with lines showing 95% CI. ED, emergency department; OR, odds ratio; CI, confidence interval; AF, atrial fibrillation.

Figure 2 shows the OR of clinical variables associated with referral to an ED in a univariate analysis. Previously diagnosed AF and prior treatment with AC or antiarrhythmic medication were inversely associated with ED referral. Arrival during the weekend was not associated with referral to an ED.

The multivariate model is shown in Table 2. Factors that were significantly associated with referral to an ED included younger age, lower SpO_2_, higher pulse rate, vascular disease, newly diagnosed AF, and evening or night shifts. Prior treatment with AC, beta blockers or antiarrhythmic medication was associated with home discharge. The variables in the model remained significant even after adjusting for the CHA_2_DS_2_-VASc score.

### 3.3. Subgroup Analysis of Patients with Newly Diagnosed AF

We performed a subgroup analysis for patients presenting with a first diagnosis of AF (*n* = 2033). In this subgroup (Table 3), patients referred to an ED had a history of vascular disease, arrived during evening or night shifts, and had lower SpO_2_. The group referred home was more likely to receive treatment with beta blockers, ACEI, or ARBs.

Table 4 shows the factors associated with referral to an ED in the multivariate analysis. Prior vascular disease and arrival during night shifts were associated with referral to an ED, whereas high SpO_2_ and prior treatment with a beta blocker or an ACEI were inversely associated with ED referral.

Among 307 patients discharged home with a newly diagnosed AF episode, 186 (60.6%) received a prescription for AC at discharge; all other patients were advised to follow up with the primary care physician to initiate AC therapy. Enoxaparin was prescribed in 29% of patients, followed by apixaban (24.4%), rivaroxaban (2.9%), warfarin (3.6%), and dabigatran (0.7%).

### 3.4. Trend Analysis

**Figure 3:**
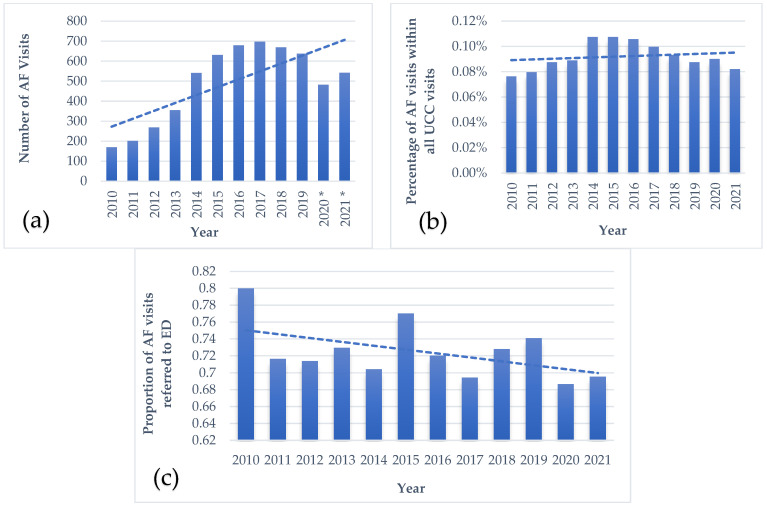
(**a**) Graphical display of the number of visits with primary diagnosis of AF per year. *p*-Value for trend = 0.011; (**b**) Graphical display of the proportion of AF visits within all visits to UCC by year. *p*-Value for trend = 0.303; (**c**) Graphical display of the proportion of patients diagnosed with AF referred to the ED by year. *p*-Value for trend = 0.050. * Indicates the years of the COVID-19 pandemic. Dashed lines indicate linear trendlines. AF, atrial fibrillation; ED, emergency department.

There was a significant rise in visits with a diagnosis of AF over the study period from 2010 until 2021 (*p* = 0.011) (Figure 3a). During the COVID-19 pandemic, there was some decline. The rate of AF diagnosis compared with other diagnoses of the total UCC visits was stable (*p* = 0.303) (Figure 3b). Over the study period, the proportion of patients with AF referred to EDs trended downwards (*p* = 0.050) (Figure 3b).

**Figure A1:**
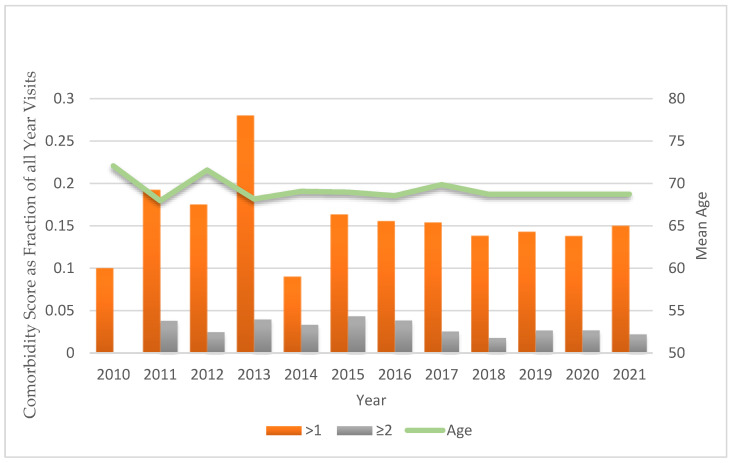
Comorbidity score and mean age over the study period per year.

In order to evaluate whether the complexity of the patients changed over the years, we compared the number of comorbidities and the mean age over the study period, and it was shown to be stable (Figure A1 in the Appendix A).

**Figure 4:**
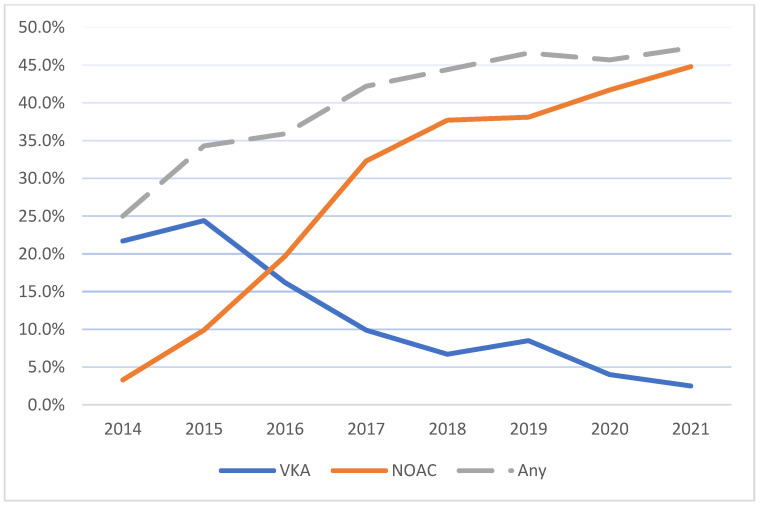
Rates of anticoagulation medications in patients with previously diagnosed AF. *p*-Value < 0.001 for all trends. NOAC, novel oral anticoagulant; VKA, vitamin K antagonist; AF, atrial fibrillation.

During 2014–2021, the rate of NOAC therapy consistently increased (*p* < 0.001) and that of VKA therapy decreased (*p* < 0.001) (Figure 4). Among the patients eligible for AC therapy with CHA_2_DS_2_-VASc score ≥ 2, the rate of AC was 61.9% in the final year of this study.

## 4. Discussion

This study provides several important findings with regard to patients presenting to UCCs with a diagnosis of AF. We showed that higher pulse, prior vascular disease, and arrival during evening or night shifts were significantly associated with referral to an ED, whereas older age, higher SpO_2_, previous diagnosis of AF, treatment with anticoagulation, beta blockers, and antiarrhythmics were inversely associated with ED referral. Second, we showed that the number of UCC visits due to AF increased while the rate of ED referrals decreased over the years. Finally, we showed a trend toward higher utilization of AC over the years, mainly NOAC.

It is estimated that approximately a third of the population will experience AF lifelong [23]. AF is a major cause of ED visits worldwide [11,21], and some of these visits are unnecessary [24]. In addition, there is growing consensus that AF can be optimally managed following discharge from an ED in outpatient settings [25,26,27,28]. Here, we showed that 27.8% of the patients were discharged home from UCCs. We believe that understanding predictors of referral to an ED may help stratify patients with AF in the community.

The CHINA-AF and ORBIT-AF investigators showed that patients with a high risk for stroke (high CHADS_2_ and CHA_2_DS_2_-VASc scores) and a history of CHF or cardiovacscular disease were significantly more likely to be hospitalized [29,30]. In our study, the CHA_2_DS_2_-VASc score was not associated with increased referrals to Eds. Prior diagnosis of AF, treatment with beta blockers, AC, and antiarrhythmics were all correlated with home discharge, emphasizing the importance of controlling symptoms and comorbidities as suggested by the ABC pathway [12,13]. Adherence to the ABC pathway, particularly long-term adherence, has been shown to reduce stroke risk and increase survival [31,32]. In contrast to our findings, the CHINA-AF and ORBIT-AF registries showed that antiarrhythmic therapy was related to an increased risk of hospitalization [29,30]. This could be explained by the nature of patients referred to UCCs who are usually deemed stable by the referring physician.

Night shifts in EDs are associated with poorer outcomes [33,34,35]. Here, we showed that patient arrival in the evening and especially during night shifts were highly predictive of ED referral. We hypothesized that this may reflect the reduced number of medical teams and the medical complexity of patients arriving during these shifts.

In our study, male patients were more likely to be referred to EDs in the univariate analysis but not in the multivariate model. In a subanalysis, we found that males had more comorbidities than females. This finding is in contrast with the results of the CHINA-AF study, which revealed a higher frequency of hospitalization among women with AF seen in EDs [36]. A 2016 Spanish study did not find a significant difference in women referred from the ED to the cardiology department [37]. In a systematic review, it was found that men had slightly higher AF hospitalization than women, yet the authors explained that this finding was inconclusive [38]. In another study, women seemed to be referred less and later in the disease course to a specialized outpatient electrophysiology clinic [39]. Similarly, data from a large Canadian cohort showed increased stroke risk, decreased referral to a cardiologist, and poorer treatment of cardiovascular risk factors in women compared with men [40]. Further research is needed to better understand sex differences in the management of acute AF.

**Figure A3:**
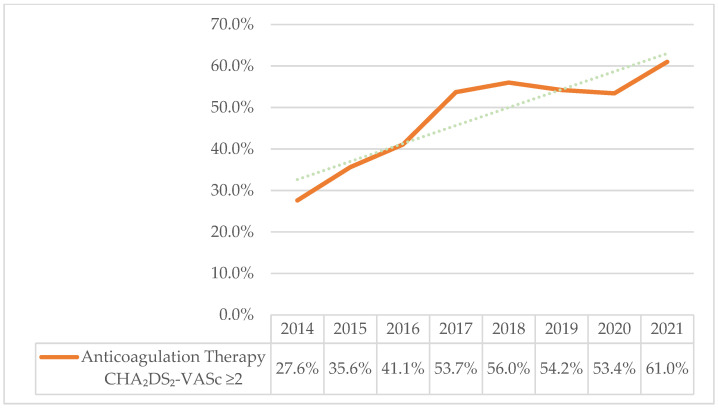
Rates of anticoagulation in patients with CHA_2_DS_2_-VASc ≥ 2 as a trend over years. *p*-Value for trend < 0.001. NOAC, novel oral anticoagulant; VKA, vitamin K antagonist; AF, atrial fibrillation. Dotted green line indicate linear trendline.

In the cohort presented, adherence to AC in CHA_2_DS_2_-VASc in eligible patients presenting to UCCs trended upwards and reached 61% in the final year (Figure A3). In prior studies from Israel, AC therapy was shown to reach almost 50% in the final years of a 2014–2015 study [41] and up to 67% in the years 2018–2019 [42]. Importantly, both studies were conducted using data from a single HMO, while our study incorporates data from all the HMOs in the country. A study in Korea showed a similar trend with 59.5% of patients receiving AC during the final year of the study in 2017 [20]. These trends are encouraging, yet AC rates remain suboptimal.

The prescription of AC in ED settings has been shown to increase patient compliance [43]. In addition, in the EMERG-AF study and in a prospective study from China, a mortality benefit was observed with this approach [14,29]. Despite this, ED prescription practices for newly diagnosed AF are often suboptimal. In two studies, only 14.5–46.7% of patients were prescribed AC [15,44]. In our study, 60.6% of patients with newly diagnosed AF were discharged home with a prescription for AC. Although this is much better than previous studies, further improvement is still needed.

In a study analyzing nationwide ED visits for AF in the United States between 2007 and 2014, the annual volume of ED visits and hospital admissions continued to increase, despite a gradual reduction in admission rates after 2011 [21]. Similarly, we showed an increase in the number of UCC visits with AF, whereas there was a significant reduction in the number of patients referred to the ED over the study period. Nevertheless, referral rates remain high. We had no data on how many patients were finally admitted to the hospital. This is an area for future research.

During 2020, there was a decrease in the number of AF visits and in referrals to the ED. We assume that the reason for this is the COVID-19 pandemic. Prior studies have shown that hospital admissions, ED, and UCC visits decreased [45,46], which is paradoxical given the rising rates of AF diagnosis in the community and during hospitalization, likely due to the arrhythmogenicity of the SARS-CoV-2 virus [47,48]. We hypothesize that patients postponed seeking medical care due to concerns of exposure.

Patients with AF are more likely to have comorbidities, and the burden of comorbidities in AF is increasing [17]. This burden has direct implications regarding morbidity and mortality risk, as highlighted in the GARFIELD-AF study [49]. It was shown to be associated with lower socioeconomic status and older age [17,50]. Indeed, in our study, patients with comorbid conditions were more likely to be referred to EDs. Interestingly, the comorbidity burden and the mean age of the patients presenting at the UCC network were stable during the study period, in contrast with population-based studies. We hypothesize that the population presenting to UCCs is younger, healthier, and has an increased rate of asymptomatic diagnosis of AF due to wearable technology, which might have diluted the effect of the increased comorbidities rate. Stratifying this specific subset of patients to outpatient care when possible may reduce the burden of AF on hospital-based care.

This study has several limitations. Despite the extensive database including a diverse population of patients, the study was conducted in a single country and may not be generalizable to other populations. Second, this is a retrospective study, and this is limited by potential inherent biases and possible confounding variables. Furthermore, the comparisons between our UCC cohort and hospital-based cohorts could be flawed because although these populations overlap; they are not the same.

In conclusion, UCCs are effective in reducing the burden of referrals of patients with AF to the ED. Optimizing AF medications and controlling comorbidities allow the management of these patients in the UCC rather than in the hospital setting, thereby assisting with overcrowding and resource-sparing. This goal may be achieved by encouraging physicians in UCCs, EDs, and the community to prescribe AC and other therapies based on the ABC pathway during the initial diagnosis of AF rather than waiting for cardiologist affirmation.

## Figures and Tables

**Table 1:** Patient characteristics by discharge destination.

Clinical Characteristic *	Overall (N = 3927)	ED (N = 2734)	Home (N = 1193)
Age	68.74 ± 14.3	68.4 ± 14.8	69.5 ± 13.1 §
Male	1837 (46.8)	(48.8)	(42.2) §
Previously diagnosed AF	1893 (48.2)	(36.8)	(74.3) §
*Comorbidities*			
CHF	143 (3.6)	(3.6)	(3.8)
Hypertension	1366 (34.8)	(36.6)	(30.6) §
Diabetes mellitus	557 (14.2)	(15.4)	(11.3) §
CVA/TIA	105 (2.7)	(2.9)	(2.1)
Vascular disease **	429 (10.9)	(12.7)	(7.0) §
CAD	416 (10.6)	(12.3)	(6.6) §
PVD	19 (0.5)	(0.6)	(0.3)
CKD	66 (1.7)	(2.0)	(1.0) §
COPD	56 (1.4)	(1.7)	(0.8) §
*Comorbidity Score*			
0	3238 (82.4)	(80.3)	(87.3)
1	581 (14.8)	(16.5)	(11.0) §
Above 2	108 (2.8)	(3.2)	(1.8) §
*Medications*			
Aspirin	290 (7.4)	(7.4)	(7.5)
Clopidogrel	55 (1.4)	(1.3)	(1.7)
Anticoagulation medication †	911 (23.2)	(15.0)	(41.9) §
VKA	222 (5.7)	(3.7)	(10.1)
Enoxaparin	11 (0.3)	(0.2)	(0.5)
Apixaban	387 (9.9)	(6.0)	(18.6) §
Dabigatran	80 (2.0)	(1.4)	(3.4) §
Rivaroxaban	145 (3.7)	(2.5)	(6.4) §
Unknown anticoagulation	74 (1.9)	(1.2)	(3.5) §
Antiarrhythmic medication ‡	318 (8.1)	(5.0)	(15.3) §
Beta blocker	698 (17.8)	(12.8)	(29.2) §
Propafenone	144 (3.7)	(2.6)	(6.1) §
Amiodarone	92 (2.3)	(1.4)	(4.6) §
Flecainide	68 (1.7)	(0.9)	(3.6) §
Digoxin	18 (0.5)	(0.2)	(1.1) §
Sotalol	18 (0.5)	(0.4)	(0.5)
ACEI	143 (3.6)	(3.3)	(4.4)
ARB	87 (2.2)	(2.0)	(2.7)
*Symptoms Onset*			
<48 h	1933 (49.2)	(49.5)	(48.5)
*Shift of Arrival*			
Morning	1392 (35.4)	(33.6)	(39.7)
Evening	1982 (50.5)	(51.3)	(48.5) §
Night	553 (14.1)	(15.1)	(11.7) §
Weekend visit	1262 (32.1)	(32.8)	(30.6)
Pulse (BPM)	103.2 ± 28.0	103.6 ± 28.6	102.0 ± 26.6
Systolic BP (mmHg)	134.6 ± 24.4	134.4 ± 25.2	135.2 ± 22.7
Temperature (°C)	36.7 ± 0.3	36.7 ± 0.4	36.7 ± 0.3 §
Hb (G/dL)	12.8 ± 2.2	12.8 ± 2.3	12.9 ± 2.0
SpO_2_ (%)	96.5 ± 3.0	96.3 ± 3.3	96.8 ± 2.2 §
SpO_2_ < 95%	567 (14.4)	(15.9)	(11.1) §

* Numbers indicate mean ± SD; numbers in parentheses indicate percentages. ** Vascular disease as described in the CHA_2_DS_2_-VASc score includes CAD, PVD, and complex aortic disease. § *p*-Value < 0.05. † Anticoagulation medication includes patients receiving chronic medical therapy with NOAC, VKA, or an unknown anticoagulant. ‡ Antiarrhythmic medication includes patients receiving chronic medical therapy with propafenone, amiodarone, flecainide, digoxin, or sotalol. ED, emergency department; CHF, congestive heart failure; CVA, cerebrovascular accident; TIA, transient ischemic attack; AF, atrial fibrillation; CAD, coronary artery disease; PVD, peripheral vascular disease; CKD, chronic kidney disease; COPD, chronic obstructive pulmonary disease; VKA, vitamin K antagonist; ACEI, angiotensin-converting-enzyme inhibitor; ARB, angiotensin receptor blocker; BPM, beats per minute; Hb, hemoglobin; BP, blood pressure; SD, standard deviation.

**Table 2:** Predictors of referral to the emergency department *—multivariate analysis.

Variable		OR	95% CI	*p*-Value
Age		0.99	0.99–1.00	0.024
Previously diagnosed AF		0.31	0.26–0.37	<0.001
SpO_2_ (%)		0.94	0.91–0.97	<0.001
Pulse		1.00	1.00–1.01	0.024
Anticoagulation medication †		0.56	0.46–0.67	<0.001
Antiarrhythmic medication ‡		0.63	0.49–0.82	<0.001
Beta blocker		0.63	0.52–0.76	<0.001
Vascular disease		1.87	1.43–2.45	<0.001
Shift of arrival	Morning	-	-	Reference
	Evening	1.31	1.11–1.55	0.001
	Night	1.68	1.32–2.15	<0.001

OR, odds ratio; CI, confidence interval. * N of patients with outcome = 2734. † Anticoagulation medication includes patients receiving chronic medical therapy with NOAC, VKA, or an unknown anticoagulant. ‡ Antiarrhythmic medication includes patients receiving chronic medical therapy with propafenone, amiodarone, flecainide, digoxin, or sotalol.

**Table 3:** Patient characteristics by discharge destination in newly diagnosed atrial fibrillation.

Clinical Characteristic *	Overall (N = 2033)	ED (N = 1726)	Home (N = 307)
Age	68.65 ± 15.07	68.55 ± 15.19	69.22 ± 14.39
Male	1020 (50.2)	(50.5)	(48.5)
*Comorbidities*			
CHF	57 (2.8)	(2.5)	(4.2)
Hypertension	780 (38.4)	(38.7)	(36.5)
Diabetes mellitus	334 (16.4)	(16.3)	(16.9)
CVA/TIA	59 (2.9)	(3.1)	(2.0)
Vascular disease **	256 (12.6)	(13.4)	(8.1) §
CAD	249 (12.2)	(13.0)	(8.1) §
PVD	12 (0.6)	(0.7)	(0.0)
CKD	34 (1.7)	(1.7)	(1.3)
COPD	40 (2.0)	(2.1)	(1.3)
*Comorbidity Score*			
0	1643 (80.8)	(80.0)	(85.0)
1	333 (16.4)	(17.0)	(13.0)
Above 2	57 (2.8)	(3.0)	(2.0)
*Medications*			
Aspirin	149 (7.3)	(7.0)	(9.4)
Clopidogrel	27 (1.3)	(1.2)	(2.0)
Anticoagulation medication †	60 (3.0)	(2.7)	(4.2)
VKA	25 (1.2)	(1.1)	(2.0)
Enoxaparin	5 (0.2)	(0.2)	(0.7)
Apixaban	12 (0.6)	(0.6)	(0.3)
Dabigatran	3 (0.1)	(0.2)	(0)
Rivaroxaban	4 (0.2)	(0.2)	(0.3)
Unknown anticoagulation	11 (0.5)	(0.5)	(1.0)
Antiarrhythmic medication ‡	8 (0.4)	(0.3)	(1.0)
Beta blocker	149 (7.3)	(6.4)	(12.7) §
Propafenone	5 (0.2)	(0.2)	(0.7)
Amiodarone	1 (0)	(0)	(0)
Flecainide	1 (0)	1 (0)	0 (0)
Digoxin	1 (0)	1 (0)	0 (0)
Sotalol	0 (0)	(0)	(0)
ACEI	56 (2.8)	(2.3)	(5.2) §
ARB	35 (1.7)	(1.4)	(3.3) §
*Symptoms Onset*			
<48 h	954 (46.9)	(47.6)	(42.7)
*Shift of Arrival*			
Morning	736 (36.2)	(35.1)	(42.7)
Evening	1022 (50.3)	(50.7)	(47.9) §
Night	275 (13.5)	(14.3)	(9.4) §
Weekend	669 (32.9)	(33.1)	(31.9)
Pulse (BPM)	102.3 ± 28.5	102.4 ± 28.8	101.3 ± 27.0
Systolic BP (mmHg)	134.7 ± 25.4	134.4 ± 25.8	136.4 ± 23.0
Temperature (°C)	36.7 ± 0.4	36.7 ± 0.4	36.7 ± 0.3
Hb (G/dL)	13.0 ± 2.2	12.9 ± 2.2	13.1 ± 2.1
SpO_2_ (%)	96.3 ± 3.4	96.2 ± 3.6	96.7 ± 2.3 §
SpO_2_ < 95%	346 (17.0)	(17.7)	(13.0) §

* Numbers indicate mean ± SD or frequency (percentage). ** Vascular disease as described in the CHA_2_DS_2_-VASc score includes CAD, PVD, and complex aortic disease. § *p*-Value < 0.05. † Anticoagulation medication includes patients receiving chronic medical therapy with NOAC, VKA, or an unknown anticoagulant. ‡ Antiarrhythmic medication includes patients receiving chronic medical therapy with propafenone, amiodarone, flecainide, digoxin, or sotalol. ED, emergency department; CHF, congestive heart failure; CVA, cerebrovascular accident; TIA, transient ischemic attack; AF, atrial fibrillation; CAD, coronary artery disease; PVD, peripheral vascular disease; CKD, chronic kidney disease; COPD, chronic obstructive pulmonary disease; VKA, vitamin K antagonist; ACEI, angiotensin-converting-enzyme inhibitor; ARB, angiotensin receptor blocker; BPM, beats per minute; Hb, hemoglobin; BP, blood pressure; SD, standard deviation.

**Table 4:** Predictors of referral to emergency department in newly diagnosed atrial fibrillation *—multivariate analysis.

Variable		OR	95% CI	*p*-Value
SpO_2_ (%)		0.95	0.91–0.99	0.027
Beta blocker		0.52	0.34–0.78	0.002
ACEI		0.45	0.24–0.84	0.013
Vascular disease		1.83	1.18–2.84	0.007
Shift of arrival	Morning	-	-	Reference
	Evening	1.28	0.99–1.67	0.065
	Night	2.01	1.29–3.13	0.002

OR, odds ratio; CI, confidence interval; ACEI, ACE inhibitor. * N of patients with outcome = 1726. Antiarrhythmic medication includes patients receiving chronic medical therapy with propafenone, amiodarone, flecainide, digoxin, or sotalol.

**Figure A2:**
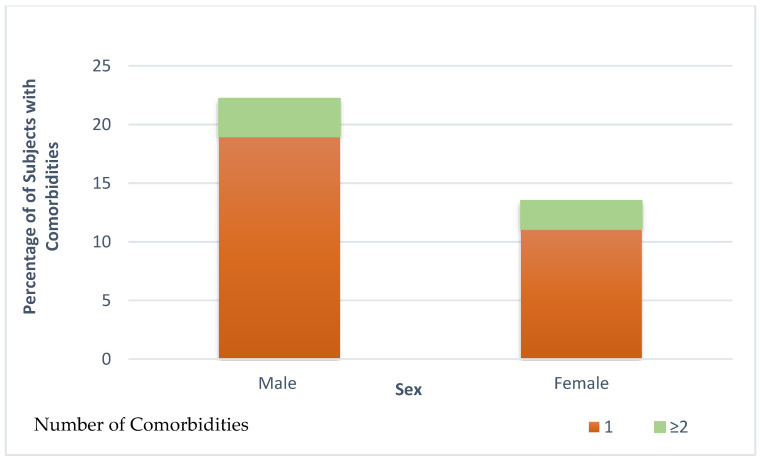
Difference between sexes and comorbidity scores.

## Data Availability

Restrictions apply to the availability of these data. Data was obtained from TEREM network and are only available with the network’s permission.

## Abbreviations

AC	Anticoagulation
ACEI	Angiotensin-converting-enzyme inhibitor
ARB	Angiotensin receptor blocker
AF	Atrial fibrillation
BP	Blood pressure
CAD	Coronary artery disease
CHF	Congestive heart failure
CKD	Chronic kidney disease
COPD	Chronic obstructive pulmonary disease
CVA	Cerebrovascular accident
ECG	Electrocardiography
ED	Emergency department
EMS	Emergency medical services
EMR	Electronic medical record
HB	Hemoglobin
HMO	Health maintenance organization
NOAC	Novel anticoagulant
OR	Odds ratio
PVD	Peripheral vascular disease
SpO_2_	Oxygen saturation measured with pulse oximetry
TIA	Transient ischemic attack
UCC	Urgent care center
VKA	Vitamin K antagonist

## Appendix A

**Table A1:** Pre-specified clinical variables for analysis.

Age
Male
Previously diagnosed AF
CHF
Hypertension
Diabetes mellitus
CVA/TIA
Vascular disease
*Medications*
Aspirin
Clopidogrel
Anticoagulation medication
Antiarrhythmic medication
Beta blocker
ACEI
ARB
Symptoms onset (<48 h)
*Shift of Arrival*
Morning
Evening
Night
Pulse (BPM)
Systolic BP (mmHg)
Temperature (°C)
SpO_2_ (%)
